# Cryptococcoma of a transplanted kidney in a patient presenting with recurrent urinary tract infection: a case report

**DOI:** 10.1186/s12882-018-0891-8

**Published:** 2018-04-23

**Authors:** Albert Z. Muranda, Ludolf Greeff, Mike M. Sathekge, Thabo Lengano, Victor O. L. Karusseit

**Affiliations:** 1 Department of Nephrology, Steve Biko Academic Hospital and The University of Pretoria, Pretoria, South Africa; 2 Department of Nuclear Medicine, Steve Biko Academic Hospital and The University of Pretoria, Pretoria, South Africa; 3 Department of Surgery, Faculty of Health Sciences, Steve Biko Academic Hospital and The University of Pretoria, Private Bag X323, Arcadia, Pretoria, 0007 South Africa

**Keywords:** Cryptococcosis, Cryptococcoma, FDG-PET/CT scan, Kidney transplant, Urinary tract infection, Case report

## Abstract

**Background:**

Cryptococcosis is an important opportunistic infection of organ transplant recipients. It is the third most common fungal infection of transplant patients and occurs especially in kidney recipients. *Cryptococcus neoformans* is a ubiquitous fungus which infects humans by inhalation of spores. *C. gattii* has more recently been recognised as a pathogen.

Infection commonly is disseminated affecting mainly the central nervous system and the lungs. Cryptococcoma, a localised form of the disease, has been described in various organs. We present a unique case of a cryptococcoma in a transplanted kidney. The lesion was not seen on ultrasound or uncontrasted computerised tomography but was detected by FDG-PET/CT.

**Case presentation:**

A 30 year old woman received a deceased donor kidney transplant in 2005. Due to chronic allograft nephropathy in 2014, cyclosporine and azathioprine immunosuppression was changed to tacrolimus and mycophenolate. After rapid deterioration of renal function in 2015 due to suspected non-adherence to immunosuppressants, steroid pulses were administered. The patient developed severe recurrent bacterial urinary tract infections and demonstrated several features of severe immunosuppression. She was treated for cytomegalovirus infection and BK virus was demonstrated in the urine. In addition, Kaposi sarcoma of the stomach was diagnosed on endoscopic biopsy. A metabolically-active lesion of the kidney transplant was imaged on FDG-PET/CT scan. Biopsy of the lesion demonstrated infection with *cryptococcus*. *Escherichia coli* with the same antibiotic sensitivity spectrum as that in the urine was cultured from the biopsy. *Cryptococcus* was not cultured from urine at that time or from several subsequent specimens. The lesion was not detected by conventional imaging. The patient manifested no other evidence of cryptococcosis. The lesion responded poorly to treatment with fluconazole.

**Conclusions:**

This is probably the first report of a case of a cryptococcoma in a transplanted organ. FDG-PET/CT scan, which is dependent on cellular metabolism, proved useful in visualising the lesion. Clinicians should be aware of this rare presentation of cryptococcosis in organ transplant recipients.

## Background

Cryptococcosis is a serious fungal infection which occurs usually in immunocompromised subjects [1]. *Cryptococcus neoformans* fungal spores are ubiquitous and commonly originate from pigeon droppings [2], but *Cryptococcus gattii* has different ecological sources [3]. Spores enter in the patient through the airway. In immunocompromised patients the disease is due to reactivation of dormant infection in most cases [4]. The most common manifestation of systemic cryptococosis is central nervous system disease in the form of meningoencephalitis, but the lungs are frequently affected [2]. A localised form of the disease, or cryptococcoma, can occur in an affected organ and has been described in unusual locations [5, 6]. This has long been recognised radiologically, and cryptoccoma must be distinguished from other granulomatous infections or malignant tumors [7]. Localised cryptococcal lesions usually occur in conjunction with systemic disease. We present a case of a cryptococcoma in a transplanted kidney in a patient with no other manifestations of cryptococcosis. The patient presented with recurrent severe bacterial urinary tract infection, for which the reason was obscure. The performance of an 18F-fluorodeoxyglucose positron emission tomography (FDG-PET/CT) revealed a metabolically-active lesion in the transplanted kidney in which cryptococcosis was identified on biopsy. A cryptococcoma in a transplanted organ has not been previously reported.

## Case presentation

A 30 year old woman was diagnosed with end stage renal disease which was suspected to be a complication of previous malarial illness. Haemodialysis was initiated in 2000 and a deceased donor kidney transplant was performed in 2005. Immunosuppression was by cyclosporin, azathioprine and prednisone, without induction therapy. The post-transplant course was stable with good renal function. The patient developed diabetes mellitus in 2010 and was placed on insulin. Parathyroidectomy was performed in 2011 for hyperparathyroidism. She was treated in hospital once in 2012 for a urinary tract infection.

**Fig. 1:**
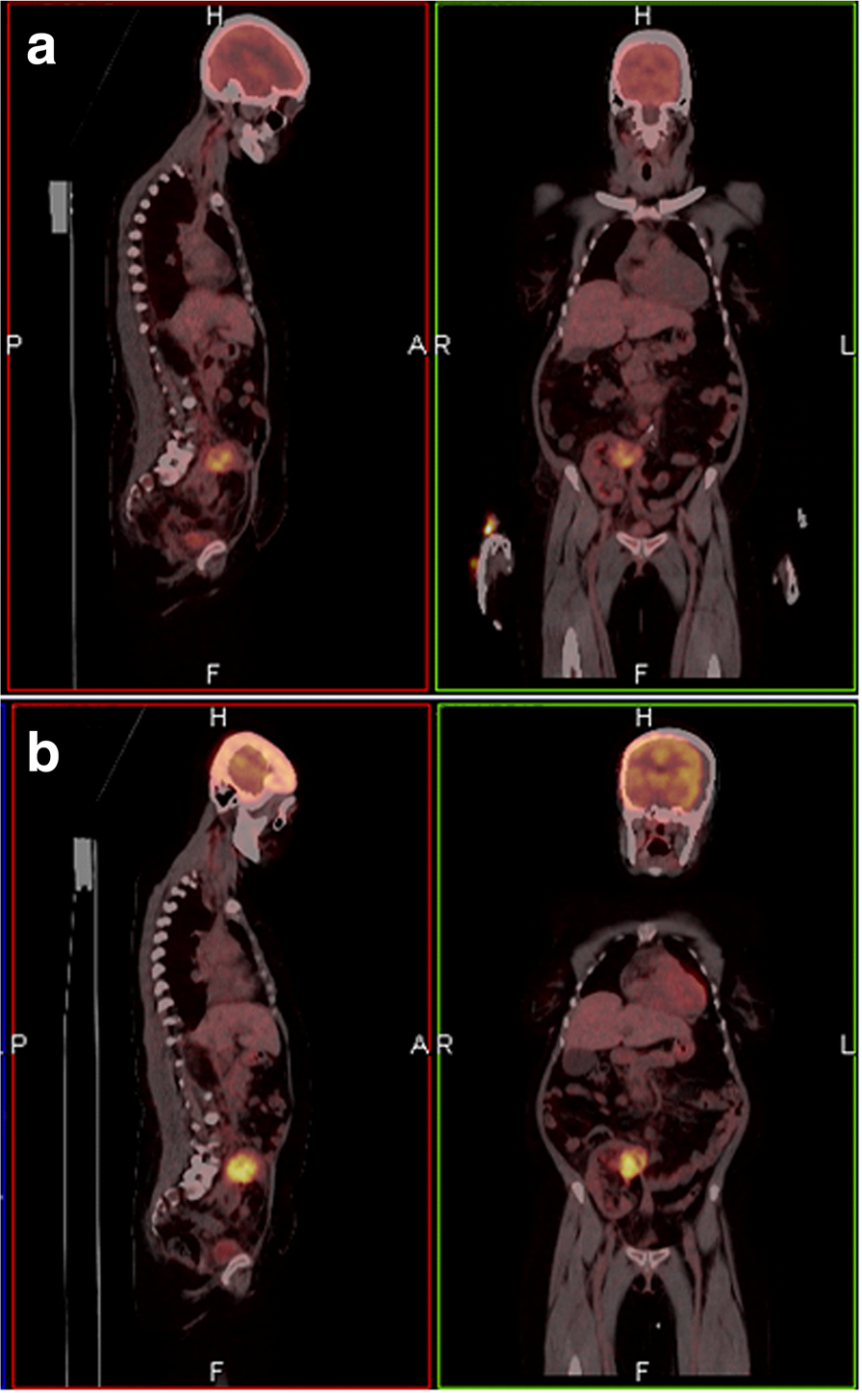
FDG-PET/CT scan demonstrating a metabolically active lesion in the transplanted kidney (panel **a**), and exacerbation on follow-up scan (panel **b**)

Renal function deteriorated over a period of 6 months in 2014, serum creatinine increasing from a baseline of less than 100 to more than 300 μmol/l. This prompted the performance of a transplant biopsy. Chronic allograft nephropathy was diagnosed and immunosuppression changed to tacrolimus (target serum trough level 5-7 ng/ml), mycophenolate mofetil (1 g twice daily) and prednisone (10 mg/day). Renal function remained stable until rapid deterioration occurred early in 2015 due to suspected non-adherence to immune suppressants during a foreign visit. Three steroid pulses (methylprednisolone 250 mg daily) were administered, and a repeat transplant biopsy was performed. The histological appearance was essentially unchanged. Renal function improved somewhat with serum creatine decreasing from an initial value of 640 μmol/l to 395 μmol/l, and then stabilising at a new baseline of about 380 μmol/l after one further dose of methylprednisone 500 mg. Subsequently viral infection due to cytomegalo-, and BK-virus occurred at different times. The virus infections were diagnosed by quantitative serum PCR for CMV and by urine PCR for BK virus. CMV infection, which presented as a febrile illness, was treated because of a sustained viral load of 250–671 copies/ml. Treatment was by induction with intravenous gancyclovir and maintenance with oral valgancyclovir. The BK viral load in the urine was 269,000 copies/ml. Immunosupression was progressively reduced to a tacrolimus target trough level of 5 ng/ml, mycophenolate mofetil 500 mg twice daily and prednisone 5 mg daily and viral disease remained quiescent. Late in 2014 and during 2015 the patient developed recurrent episodes of severe bacterial urinary tract infection which were accompanied by SIRS response. She was admitted to hospital on five occasions with intervals of one to 2 months. The infections responded each time to empiric (usually amoxicillin/clavulanic acid) and/or directed (on occasion switched to a carbapenem) antibiotic therapy for 10–14 days. Initially *Klebsiella pneumoniae* was cultured from urine and blood, but on the last 3 occasions *Escherichia coli (E. coli)* was cultured, each time with similar antibiotic sensitivity. Ultrasound of the transplant, vesicocystourography and cystoscopy were non-contributory to the causation of recurrent infection apart from grade 2 reflux into the transplant ureter. Various prophylactic antibacterials were prescribed without success. On each occasion of admission for sepsis the graft was tender and it was decided to perform an FDG-PET/CT scan. This revealed a metabolically active lesion in the upper pole of the transplanted kidney, suggestive of an abscess (Fig. 1a). Attempted aspiration of the lesion did not yield pus after several passes, and was followed by a core needle biopsy. The biopsy yielded poor non-diagnostic tissue, but a positive culture of an *E. coli* with the same antibiotic sensitivity spectrum as that cultivated from urine and blood. The biopsy was repeated and this time yielded diagnosable material. Routine histological sections were stained with haematoxylin and eosin and revealed renal tissue with a prominent infiltrate of *cryptococcus* round yeast bodies. The fungal elements were organised into groups in a myxoid and inflammatory background in most of the tissue. Alcian blue staining demonstrated a thick mucinous fungal capsule. The microscopy confirmed the presence of chronic allograft nephropathy. There were areas of prominent interstitial fibrosis with atrophic tubuli and occasional sclerotic glomeruli. There were no specific features of BK virus nephropathy present. The biopsies had not been specifically cultured for fungal pathogens as the finding was unexpected. At the time of discarding the plates at 48 h, there had been no fungal growth.

On the basis of the radiological and histological appearances, a diagnosis of cryptococcoma of the transplanted kidney was made. Investigation for systemic cryptococcosis was commenced. An uncontrasted brain and lung CT scan was normal. Cerebrospinal fluid examination yielded the following: glucose 3.6 mmol/l, protein 0.29 g/l and adenosine deaminase 1.5 u/l. There were no cells present. India ink staining and *cryptococcus* latex antigen test (CLAT) of the fluid were negative. Bacterial and fungal culture were negative. The lungs were examined clinically, and radiographically by plain X-ray and CT scan. They were found to be normal. The serum *cryptococcus* latex antigen test (s-CLAT) was negative. Multiple subsequent blood and urine specimens were negative for fungal culture.

The patient was treated with fluconazole 400 mg daily with the intention of continuing for 6 to 12 months. During this time the patient remained chronically ill with nausea, anorexia and loss of weight, as well as the recurrent urinary tract infections. On follow-up FDG-PET/CT scan after 2 months the cryptococcoma showed a significant increase in size and intensity (Fig. 1b). A gastroscopy was performed for the upper gastro-intestinal symptoms. A mucosal mass was seen, biopsy of which revealed Kaposi sarcoma.

Throughout her protracted illness the patient remained unwilling to accept any reduction of the immunosuppression for fear of losing the kidney. Eventually, in light of poor renal function and life-threatening infections, she acceded to reduction and cessation of immunosuppressants, and was started on haemodialysis. Treatment for the cryptococcosis was escalated by adding 200 mg of fluconazole after each dialysis session. The lesion in the kidney which had become detectable on ultrasound was apparently unchanged. The patient died soon after initiation of dialysis during admission to hospital for an episode of severe sepsis.

## Discussion

This is the first description of a cryptococcoma occurring in a transplanted organ. Cryptococcoma is a specific form of cryptococcal disease manifesting histologically as a localised inflammatory mass containing a myriad of *cryptococcus* organisms [8]. Cryptococcomas occur mainly in the central nervous system and the lungs where they must be distinguished from granulomatous infections and tumours [9, 10].

This case of cryptococcal disease is most likely due to the effects of immunosuppression. Donor transmission is unlikely given that the disease occurred 10 years after the kidney transplant [11]. It occurred during a period of intensified immunosuppression by switching to a combination of tacrolimus and mycophenolate mofetil. In addition a course of pulsed steroids had been administered 2 months before the diagnosis of the lesion in the kidney.

Only candidiasis and aspergillosis are more frequent causes of fungal infection than cryptococcosis in organ transplant recipients [1]. The overall incidence of cryptococcosis is about 3% in solid organ recipients but is relatively more common in kidney recipients [1]. The majority of patients have disseminated disease affecting mainly the central nervous system and lungs, but a wide variety of organs can be individually affected. Infection occurs commonly 2 to 5 years after transplantation. Patients taking calcineurin inhibitors are more likely to have disease limited to the lungs [12]. *Cryptococcus neoformans*, which occurs worldwide, is the most common cause, but *C. gattii* which occurs mainly in the tropics and subtropics, has now also been reported to cause infection in transplant recipients, especially in northwest United Stated and British Columbia in Canada [4]. Severity of cryptococcosis is related to the net state of the patient’s immunosuppression and overall survival of transplant patients is 70–80% [4].

Cryptococcoma, as a localised form of cryptococcal disease, is believed to occur more frequently in *Cryptococcus gattii* infection because the fungus secretes products that inhibit the accumulation and infiltration of infected tissues by leukocytes [13]. This may allow localised survival and proliferation of organisms resulting in a mass lesion. This is apparent in the histology of these lesions in which neutrophil leukocytes are virually absent, and histiocytes are predominant [8]. A negative s-CLAT test, as in this case, is ascribed to the localised nature of the lesion, in which the burden of organisms is relatively low [1].

Both solitary and disseminated cryptococcal disease have previously been demonstrated on FDG-PET/CT scanning [7]. This was used in this case because ultrasound was non-contributory and contrasted CT scan was contra-indicated because of deteriorating renal function. FDG-PET/CT, in which imaging is uniquely dependant on cellular metabolism, can detect malignant, inflammatory and infectious foci which may not be detected by other modalities. This includes fungal lesions in which FDG-PET/CT scanning also provides information on therapeutic response [14], which in this case was poor.

Clinical exacerbation of fungal disease can occur in immunocompromised hosts in the form of the immune reconstitution syndrome (IRS) on restoration of host immunity [15]. IRS can also occur on reduction of immunosuppression in organ transplant recipients, including some with cryptococcosis [16]. IRS is due to a transient escalation of the inflammatory response and can be difficult to differentiate from progression of infection. It is especially relevant in meningoencephalitis because of occurrence in the rigid confines of the skull. IRS seems unlikely in this case because of the prolonged course of the illness in which deterioration of the inflammatory mass on FDG-PET/CT scan occurred over a period of several months. In addition, local transplant manifestations of disease, such as the tenderness, were probably more related to the recurrent bouts of bacterial pyelonephritis.

The major burden of illness in this patient was severe relapsing bacterial pyelonephritis. Several factors predisposed to this course. Intensified immunosuppression, diabetes mellitus, and vesico-ureteric reflux were probably all contributory. Nevertheless, it seems that the fungal and bacterial infections coexisted in the kidney transplant.

One can speculate that the crytococcoma may have been causative of recurrent pyelonephritis in this patient. The effect of *cryptococcus* on leukocytes described above may have caused persistence of bacteria in the renal tissue in and around the cryptococcoma by leukocyte exclusion, and would be a novel mechanism of persistence of bacterial infection. This may be the reason for culture of *E. coli* from the first percutaneous biopsy before the successful core biopsy. On the other hand the mass effect of the cryptococcoma may have impeded drainage of urine from one or more calyces causing stasis and persistence of bacteria, and consequently recurrent bouts of urinary tract infection. While the proposition that the cryptococcoma played a role in the relapsing bacterial infection in this patient is unproven, it remains an intriguing hypothesis.

## Conclusions

In conclusion, it seems that cryptococcal infection may rarely present as a localised infection of the graft in renal transplant recipients. Clinicians should be aware of this rare presentation, and of the possibility in nonresponsive transplant infection.
